# Unsettling the treatment imperative? Chemotherapy decision‐making in the wake of genomic techniques

**DOI:** 10.1111/1467-9566.13637

**Published:** 2023-03-25

**Authors:** Emily Ross, Anne Kerr, Julia Swallow, Choon Key Chekar, Sarah Cunningham‐Burley

**Affiliations:** ^1^ Department of Sociological Studies University of Sheffield Sheffield UK; ^2^ School of Social and Political Sciences University of Glasgow Glasgow UK; ^3^ Centre for Biomedicine, Self, and Society Usher Institute University of Edinburgh Edinburgh UK; ^4^ Faculty of Health & Medicine Lancaster University Lancaster UK; ^5^ Usher Institute University of Edinburgh Edinburgh UK

**Keywords:** breast cancer, chemotherapy, gene expression profiling, genomic medicine, treatment decision‐making, treatment imperative

## Abstract

Social scientists have argued that a treatment imperative shapes experiences of biomedicine. This is evident within oncology, where discourses of hope are tempered by persistent fears surrounding cancer. It is within this context that genomic decision‐making tools are entering routine care. These may indicate that a treatment is not appropriate for a particular disease profile. We draw on qualitative interviews and observations centred on gene expression profiling to consider the implications of this technique for the treatment imperative in early breast cancer. Influenced by sociological perspectives on medical technologies, we discuss how fallibilities of established tools have forged a space for the introduction of genomic testing into chemotherapy decision‐making. We demonstrate how high expectations shaped patients’ interpretations of this tool as facilitating the ‘right’ treatment choice. We then unpick these accounts, highlighting the complex relationship between gene expression profiling and treatment decision‐making. We argue that anticipations for genomic testing to provide certainty in treatment choice must account for the sociocultural and organisational contexts in which it is used, including the powerful entwinement of chemotherapy and cancer. Our research has implications for sociological perspectives on treatment decision‐making and clinical expectations for genomic medicine to resolve the ‘problem’ of overtreatment.

## INTRODUCTION

A consequence of bioscientific development has been the expansion of treatment options available to patients and the need to negotiate a range of test results, prognostic predictions and professional recommendations in treatment decision‐making. Sociologists have shown that the shifting landscape in which patients make individual treatment choices is shaped by a wider ‘imperative to treat’, notably in cancer where discourses of positivity and hope proliferate (del Vecchio Good et al., 1990). In recent years, genomic analysis of tumour profiles has begun to drive research investment in oncology, and these techniques are becoming visible within the UK National Health Service (NHS) (Day et al., 2017; Kerr et al., 2021). Genomic analyses may determine that an available treatment is unsuitable for a particular cancer type according to its molecular profile, or that an intervention’s side effects outweigh its potential benefits. In this article, we ask how techniques aiming to personalise care, which may involve a recommendation to *decline* a specific treatment, may be shaping patient experiences of the treatment imperative. We attend to this through qualitative accounts of gene expression profiling in early breast cancer. We show that despite appearing to render treatment refusal an acceptable choice, gene expression profiling does not entirely mitigate the treatment imperative. Though the technique could provide temporary relief and moments of certainty, the treatment imperative remained strong with implications for care pathways and longer‐term reflections on cancer. We argue that anticipations for genomic tools to transform treatment decision‐making must appreciate wider disease contexts. This research draws on and extends sociological perspectives on medical technologies and treatment decision‐making.

### The treatment imperative in cancer care

A hallmark of contemporary processes of biomedicalisation is an orientation towards the future, entailing efforts to predict and prevent threats to health (Clarke et al., 2010). As well as describing the impacts of this for the organisation of medicine (Kreiner & Hunt, 2014) and patient embodiment (Sulik, 2009), sociologists have considered its consequences for experiences of treatment. In their study of cardiac interventions in later life, Shim et al. (2006) note that the drive within medicine to monitor and reduce health risks supports a ‘culturally pervasive sense of medical possibility’ (p. 480). This has resulted in doctors and patients experiencing an ‘imperative’ to treat disease at all ages and stages. Clinicians interviewed by Shim et al. experienced an ‘almost inexorable momentum towards treatment’, which they discussed in terms of a clinical standard of care as well as an ethical responsibility (2008, p. 344). Spencer et al. (2022) show how this treatment imperative is generated beyond clinical encounters. Attending to experiences of late‐life palliative care, they demonstrate its co‐production through diverse actors and circumstances including care‐providers and family members, as well as treatment schedules, biographical histories and test results. Treatment imperatives thus permeate across domains, generating ‘multiple routes of influence’ on patients’ medical decision‐making (p. 795). Through the concept of the treatment imperative, these authors show how future orientations in biomedicine facilitate the routinisation of treatment at older ages and later stages of the disease, shifting the limits of medical knowledge and of life itself (Shim et al., 2006, see also Baszanger, 2012).

The imperative to treat has often been examined by social scientists of later and end‐of‐life care (e.g. Borgstrom et al., 2020; Tate, 2022), but it is also demonstrable across oncology. Cancer research, treatment and support emanate messages of hope for individual patients (del Vecchio Good et al., 1990). This holds true across diverse cancer types, which in recent years have seen the introduction of genomic analysis and expanding knowledge of subtypes amenable to targeted treatment.^1^ A culture of experimentation, appreciation of the heterogeneity of the disease and proliferation of treatment options has shifted understandings of cancer, redefining some forms as chronic illness and extending the parameters of a ‘treatable’ disease (Baszanger, 2012; Keating et al., 2016). The biopharmaceutical industry is also an important player and driver of the expanding use of medicines (Davis, 2015), tying individual patients’ desires for a cure together with the broader social and economic forces of biomedicine in a ‘political economy of hope’ (del Vecchio Good et al., 1990; Mrig & Spencer, 2018). Within this wider culture of investment and anticipation for cure, cancer patients are ‘presented with both a presumptive obligation and imperative to “choose to live”’ (Steinberg, 2015, p. 129). As a patient herself, Steinberg writes that this leaves ‘no room for a choice to refuse treatment… The willingness to undergo treatment’s ‘cutting edge’ takes on a talismanic power’, promising not so much freedom from cancer but a ‘moral standing and recognition as an edifying subject’. In Steinberg’s experience, the treatment imperative is thus multi‐layered, tied to personal hopes for cure but also expectations of the ‘good’ cancer patient (Steinberg, 2015, p. 130). This drive towards treatment endures despite the potential for heavy side effects, with these particularly associated with chemotherapy.

Chemotherapy, the systemic administration of anticancer drugs, is sometimes used curatively but most often used to slow the progression of disease or prevent its recurrence (known as adjuvant). Chemotherapy occupies a commanding role in contemporary narratives of cancer. The strong imagery of hair loss, sickness and toxicity abound in media representations and familial memory, with suffering perceived as a necessary indicator of treatment success (Bell, 2009). So pervasive is the association between cancer treatment and chemotherapy that in one study, patients represented the refusal of adjuvant chemotherapy as ‘doing nothing’, despite receiving other interventions including surgery and hormone therapy (Charles et al., 1998). The strong imperative to treat, coupled with the cultural entwinement of cancer treatment with chemotherapy, can leave patients feeling as if they have ‘no choice’ but to undergo the treatment, even when designated as at low risk of cancer recurrence (Charles et al., 1998). The unease around forgoing chemotherapy has also been voiced by oncologists. In their study of gene expression profiling techniques, clinicians interviewed by Bourgain et al. (2020, p. 5) described that, in the context of a ‘dose culture’ where oncologists are trained to ‘believe in chemotherapy’, treatment decision‐making in the case of low‐risk breast cancers could be particularly uncomfortable. Such work, which considers the treatment imperative in early breast cancer, provides a useful counterpoint to studies considering the advanced disease. Unlike later life conditions, the harms of treatment in early breast cancer can be more acute than the disease itself, including its risk of recurrence. The ‘pervasive sense of medicine’s possibilities’ described by Shim et al. (2006) may therefore be constrained, resulting in a more complex engagement with the imperative to treat. Such complexities are becoming more pronounced in sociological studies of cancer as they begin to address the biological heterogeneity of the disease, along with its implications for diagnostic processes, treatment pathways, research involvement and thus patient experience (e.g. Ackerman, 2022; Viney et al., 2022).

### Sociological perspectives on personalising treatment

The imperative towards treatment articulated by patients and doctors alike is judged by some to be partly responsible for the current ‘problem of overtreatment’ in oncology (Pak & Morrow, 2022). Tackling overtreatment, whereby patients undergo therapy despite having minimal potential to benefit, has been cited as a key aim for the introduction of genomic techniques into cancer management (Katz et al., 2018). The use of genomic information to develop more precise or personalised diagnostic and treatment pathways is becoming more visible within health systems. In NHS breast cancer care, gene expression profiling can be deployed to aid treatment decision‐making in some early‐stage cancers. This technique uses genomic analysis to generate an estimation of the potential benefit of chemotherapy in preventing recurrence. If the benefit is deemed low, the clinical recommendation may be that the patient decline chemotherapy. A host of clinical studies have considered the impact of this technique, generally finding it influential in patients’ decisions about chemotherapy (e.g. Loncaster et al., 2017; Marshall et al., 2016). Katz et al. (2018) argue that by providing a more ‘accurate’ assessment of disease, genomic techniques provide the ‘tools a clinician needs to recommend against a treatment that does more harm than good’ (p. 1092). Within this argument, overtreatment is linked to inadequate information about the disease and resulting uncertainty about how to act. Sociological and clinical perspectives have shown that uncertainty can provoke fear for practitioners and patients and an imperative to ‘do more instead of less’ in order to reduce clinical unknowns (Heath, 2014, p. 3). In the above quote, the clinician’s access to more (refined) information is portrayed as key to ensuring that patients receive the most appropriate treatment, including its avoidance. However, this position does not account for patient engagement with genomically‐derived recommendations and how these inform decision‐making, with this dependent upon a range of individual and wider social factors. Indeed, sociologists have already questioned the extent to which decision tools alone can tackle overtreatment, calling for attention to the systems and wider contexts in which they are situated which may constrain professionals’ and patients’ abilities to ‘do less rather than more’ (Armstrong, 2021, p. 58). In this article, we add to these discussions by considering the sociocultural contexts and relationships shaping patient engagement with gene expression profiling.

Genomic medicine is widely touted as having the potential to ‘revolutionise’ the delivery of care (Samuel & Farsides, 2017), in part by providing information about the disease causing genetic variants and possible (future) treatments. However, sociologists have shown that genomic testing can exacerbate uncertainty, for example, by generating data that is not clinically actionable (Timmermans et al., 2017). This can require unexpected interpretive work for patients but also health‐care professionals as they manage patient expectations (Kerr et al., 2019). Continued uncertainty can be emotionally difficult for patients when these ‘advanced’ and ‘comprehensive’ techniques are often viewed as a last resort (Timmermans et al., 2017). From the perspective of clinicians, the uncertainties introduced by ‘imperfect’ genomic techniques require new forms of work to negotiate these technologies with clinical judgement, wider professional teams and the wishes of patients (Bourgain et al., 2020; Bourret et al., 2011).

Though the interpretation of genomic test results and their integration into the daily work of clinical practice has been explored, sociological insight is required into how patient experiences of the imperative to treat cancer might shift, as techniques aiming to *reduce* treatment become routinised. In this article, we analyse patient and clinician accounts of gene expression profiling in the UK NHS to consider its role in chemotherapy decision‐making. We reflect on the implications of this technique for the pervasive imperative to treat in oncology. We are informed by sociological approaches that see technologies, care practices, bodies and identities as continually and mutually shaped (Casper & Morrison, 2010; Timmermans & Berg, 2003). This includes clinical decisions such as chemotherapy choices, which we understand not as discrete events, but as the ongoing orchestration of a diverse range of actors and practices (Berg, 1997). We align with approaches that attend to the range of technological actors, cultural scripts and procedures that come together to materialise particular figurations of cancers: For example, as demanding ‘active’ treatment, simultaneously producing a ‘passive’ alternative (Kazimierczak, 2018). Conceptualising clinical interactions in this way draws attention to how treatment decision‐making is *generative* of ‘objects and meanings, bodies and identities’ (Kazimierczak, 2018, p. 198), including the diseases experienced by patients and paths to action available to them and their clinicians.

In what follows, we introduce our methods and analysis before discussing the emotions, relationships, technologies and practices that constitute chemotherapy decision‐making as particularly difficult for clinicians and patients. We then move to patient accounts of gene expression profiling, which appeared to alleviate the orientation towards treatment. Engaging with these accounts in depth, we then demonstrate the complexities involved in negotiating the treatment imperative. Patients’ experiences were impacted by the pervasive notion of a ‘correct’ treatment choice, varied over time, and remained situated within powerful cultural narratives of cancer and chemotherapy. As we will show, though gene expression profiling allowed some participants to decline chemotherapy, the treatment imperative remained powerful, shaping experiences of further treatment decision‐making and reflections on cancer’s return. Our research points to the ongoing discomfort felt by clinicians and patients at the separation of cancer and chemotherapy and questions the extent to which the treatment imperative in oncology can be disrupted by novel genomic techniques.

## METHODS

This article presents data from a multi‐sited project centring on patient and practitioner experiences of genomic technologies in cancer care and research. Following institutional and NHS ethical approval (NHS reference 16/YH/0229), over 200 qualitative interviews and observations were conducted for the wider study. These took place with people affected by cancer, family members, charity representatives and a range of health‐care professionals including nurses, oncologists and pathologists (Chekar et al., 2022). The findings discussed below are taken from observations and interviews on the use of gene expression profiling in standard breast cancer practice. The most established gene expression tool used in the UK NHS is named Oncotype DX, and we use the terms interchangeably throughout.

As an example of a relatively novel technique (first approved for NHS use in 2013), gene expression profiling represented a suitable case through which to explore practitioner and patient engagement with cancer genomics as ‘routine’. The technique is now used across the UK NHS but only available to breast cancer patients diagnosed with oestrogen receptor‐positive, human epidermal growth factor receptor 2‐negative and lymph node‐negative breast cancers. Additionally, these patients must be judged at intermediate risk of recurrence according to established tools used to estimate long term survival (National Institute for Health and Care Excellence, 2018). These include algorithmic tools such as *NHS Predict*. Gene expression profiling is therefore used with only a fraction of the approximately 55,000 breast cancer diagnoses in the UK each year (Crolley et al., 2020). This impacted the recruitment of clinicians and patients for our research. Data on UK usage are difficult to obtain, with this varying between sites (Crolley et al., 2020) and amongst clinicians. For example, whilst one of our Scottish oncologists described using Oncotype DX a total of ‘five or six’ times, another described referring ‘about 80’ in its first year. Between May 2017 and August 2018, we recruited and interviewed nine health‐care practitioners with experience of Oncotype DX. We interviewed 18 patients (June 2017–August 2019) who had experienced the technique as part of their cancer care at NHS sites within England and Scotland. We also conducted four observations (June–July 2017) of consultations where patients and an accompanying family member discussed treatment decisions following gene expression profiling. Online forum discussions of the technique were analysed alongside the interviews, with this work reported elsewhere (Ross et al., 2019).

Interviews with all participants were semi‐structured. Practitioner interviews covered experience with gene expression profiling, making sense of results, reporting results to patients, the impact of the technique on day‐to‐day practice and expectations for its future role. These were designed to be shorter, lasting approximately 45 min. Patient interviews began by seeking a narrative account of the interviewee’s path to diagnosis. Interviews then covered their experiences with gene expression profiling specifically, including how the technique was explained by their clinician, any independent research they had undertaken, how they made sense of their Oncotype DX result, subsequent treatment decision‐making and ongoing legacies of the result. Interviews with patients lasted between 45 min and 2 h. All interviews were audio‐recorded and transcribed verbatim. The four observations involved a researcher taking fieldnotes during and after consultations where treatment decisions were formalised following a patient’s result.

Transcripts and fieldnotes were shared between the research team and discussed with reference to the wider set of interviews and observations and to social scientific literature. During this process, it became clear how emotional chemotherapy decision‐making was for patients and that engagements with gene expression profiling were heavily shaped by the difficulties of this decision. The imperative towards treatment in cancer, linked to discourses of hope and positivity, was developed as a theme. Our analysis also led to questions about why the test was discussed as so influential in chemotherapy choices and a revisiting of social scientific literature around clinical decision‐making technologies in practice. The data were considered in light of this literature, employing techniques of constant comparison (Charmaz, 2006) in the development of key themes. This analytical work complemented and extended our existing work on gene expression profiling. Other publications have focussed on experiences of the technique as shared and discussed in online interactions (Ross et al., 2019), patient experiences of breast cancer diagnosis (Ross et al., 2021) and representations of gene expression profiling in policy and practice (Kerr et al., 2021).

## FINDINGS

### The treatment imperative in early‐stage cancer: Making the ‘right’ chemotherapy choice

Our participants’ cancers had generally been positioned as ‘good’ in a clinical setting (Kazimierczak & Skea, 2015), described as ‘small’ (Alice) and ‘treatable’ (Lois). The majority of interviewees had initially been given a positive outlook and prognosis, with treatment following well‐established clinical pathways. As described by Jane, “when you do the cancer journey, as it were, a lot of the way, you do get kind of told what to do”. Participants had experienced one or more biopsies and surgery, with most undergoing a less invasive lumpectomy rather than mastectomy. This was followed by an examination of the tumour by a pathologist to determine the cancer’s stage and hormone receptor status, which often indicates a corresponding treatment response. However, following surgery, our interviewees encountered treatment uncertainty. The prognostic algorithms used to determine a chemotherapy recommendation positioned them as at intermediate risk of cancer recurrence, described as a ‘grey area’. At this stage our patient interviewees became more explicitly enroled in treatment decision‐making. One medical oncologist described this situation pragmatically:

If [chemotherapy benefit]’s kind of borderline, then we would have a discussion with the patient about the pros and cons and then their preferences would really come into the decision.

(Medical oncologist 5)

In contrast to the straightforward discussion of preferences depicted in this quote, many of the patients we interviewed experienced such discussions as extremely challenging. They described this as their clinician “laying the decision at your door” (Dette) and feeling that it was “[their] choice whether to do it or not” (Valerie). For many, this choice was a heavy burden and extremely difficult to navigate. Though the women interviewed were generally ‘engaged patients’ (Timmermans, 2020), some of whom had confidently participated in decisions about surgery, on the issue of chemotherapy the deferral to their preferences was troubling. The decision being put to them was highly affective for interviewees, invoking vivid imagery and painful memories of family members ‘struggling’ with this ‘dehumanising’ treatment (Susan, Bethany). Though participants discussed fears surrounding chemotherapy, they also feared the return of cancer. All described a willingness to accept the treatment, if necessary, to reduce the risk of cancer’s recurrence. Some did not expect to have the option to avoid chemotherapy, as Dette explained: “everybody knows that chemotherapy gets rid of [cancer], that has to be for me then”. Discussing their initial feelings about this decision, most women described an imperative to proceed to the treatment, with four interviewees labelling this a “belt and braces” approach:

My feeling at that point was to go for the belt‐and‐braces approach, yeah, I’ll have chemotherapy. I’d rather feel sure that I’d done everything I could, um, you know, rather than be unsure of whether I’d made the right decision.

(Jane)

These initial responses were also shaped by discussions with friends and family members, some of whom voiced their own discomfort about interviewees rejecting chemotherapy:

[My partner] said to me “just take the chemotherapy because you don’t know, you know, how can you make a decision based on [not] knowing. Just take the chemotherapy.” …Even though I didn’t want to, once that little seed of doubt was planted that you had a choice, I thought “well, OK then, I will”.

(Dette)

Though participants did not want to have to undergo its physical toll, in the face of clinical uncertainty, they felt an imperative to proceed to chemotherapy. In one observation, the patient described that this decision located her between a “rock and a hard place”, lamenting that she had “always been an unlucky person” (Observation 4, July 2017). Alice explained this feeling in more depth:

I thought, oh if I don't do it, then am I making [the cancer] worse? And if I do do it, am I, you know, using a sledgehammer to crack a nut, kind of thing. How, how bad is it? And nobody was able to kind of, you know, tell me. So it was really my decision.

(Alice)

The image of chemotherapy as a sledgehammer is symbolic of women’s hesitation about proceeding to the treatment. Following a diagnosis of a ‘good’ cancer, with many having experienced minimally invasive surgeries, the prospect of weeks of a demanding treatment of uncertain benefit was hard to face. The questions posed by Alice, to which she was unable to obtain answers from her health‐care professionals, also point to a theme articulated by others; the idea that there was a ‘right’ (and thus a ‘wrong’) treatment decision to be made. This framing is also evident in clinical literature. For example, national guidance on gene expression profiling represents the technique as allowing patients to avoid ‘unnecessary treatment’ and “confirm whether their risk is correct” (National Institute for Health and Care Excellence, 2018, p. 6). However, as discussed by Shim et al. (2008), the cultural and institutional orientation towards treatment obscures the fact that risk remains, whichever pathway is chosen. This is more salient when considering the heterogeneity and instability of cancer. As one clinician explained:

There might be a limit as to how much predictive information you can get just from the cancer. Because the entirety of what’s going to happen in that patient’s next ten years is not just encoded in the cancer… that’s not going to be captured in an Oncotype test.

(Medical oncologist 1)

Nevertheless, the notion of a correct treatment choice was articulated by most interviewees. Lois was concerned that she might have chemotherapy and then “find out at a later date that I shouldn’t have had it or didn’t really need it”. The interpretation of a ‘right’ decision was also shaped by previous steps on their diagnostic journey, where uncertainties had been elided through a clear direction with regards to treatment. The complexities of cancer management and its unpredictability had previously been subsumed through categorisation of the cancer according to measurable characteristics including its size, grade and tumour markers. During the initial phases of their care pathway, though not always immediately apparent, these had corresponded to a “clear line of action to take” (Berg, 1997, p. 505). Now, interviewees became thrust into a situation of uncertainty—a situation created by the inability of established clinical tools to provide a clear answer regarding chemotherapy benefit. With so much at stake, interviewees described feeling ‘unqualified’ (Julie) to bear the responsibility for this choice, contrasting their decision‐making abilities with their clinicians’ who “see this 20 times a day” (Valerie), and questioning the utility of their ‘preferences’ in this critical situation (Zoe).

By attending to these diverse actors and practices, we have shown how (failures of) established clinical tools, patient biographies and cultural narratives of cancer came together to shape the treatment options available to our interviewees with early‐stage breast cancer. In the face of uncertainty, many suggested ascribing to the treatment imperative, to ‘do all they could’ to fight the disease (Charles et al., 1998). For both clinicians and patients, the discomfort of chemotherapy decision‐making in early cancer created a need for reassurance and a space for further decision‐making tools (Bourgain et al., 2020). In the next section, we show that gene expression profiling was thus welcomed by interviewees and how its introduction shaped their orientations towards treatment.

### A ‘state of the art’ technique: Unsettling the treatment imperative

As outlined above, novel genomic techniques are presented as having the ability to resolve clinical uncertainties, where established techniques have failed. This was attested to by the clinicians we interviewed, including one oncologist who relayed his explanation of gene expression profiling to patients:

[I] say that, um, it’s a slightly newer technology. And in addition to … tumour size, grades, nodes, which we’ll just have discussed with the patient. Then I’ll say something along the lines of “this can actually look inside the cancer cells in more detail and look at the activity of genes and proteins inside the cancer, and can give extra information over and above the things that we know about already”.

(Medical oncologist 1)

During one observation, a consultant discussed the value of this information in terms of specificity, as providing more tailored data than the ‘rough averages’ generated by prognostic algorithms (Observation 3, July 2017). These algorithms use statistical models and population data to estimate the survival benefit of adjuvant chemotherapy to individual patients. Clinicians described incorporating the Oncotype DX result alongside the data they already had about the tumour, including that generated by the algorithmic tool, using the result to “refine our risk estimate” (Medical oncologist 5). However, several participants interpreted the information provided by gene expression profiling as *surpassing* that provided by previous diagnostic techniques. Its analysis of tumour DNA was described by some women as providing a “more specific or more individualised” (Julie) assessment of chemotherapy benefit. Hazel understood that Oncotype DX addressed whether ‘for your particular genomic assay of cancer, is chemotherapy the best thing to target it?’. These women perceived Oncotype DX as ‘advanced’ (Elisa) or ‘state of the art’ (Bethany), reflecting the wider rhetoric of hope shaping the development and engagement with novel techniques in oncology (del Vecchio Good et al., 1990; Haase et al., 2015). Participants largely welcomed Oncotype DX, interpreting it as providing the information they required to make the ‘right decision’ about chemotherapy:

It’s a test which sort of sounded really good, and it would give a definitive answer about whether I needed to go for chemotherapy.

(Felicity)

I need certainty. I said, right, I'll pay for this test if necessary, to make me make the right decision.

(Alice)

Here, Alice links the specificity of gene expression profiling to certainty. For many patients (though not all), Oncotype DX was portrayed as a final ‘layer’ of diagnostic information because it resolved ambiguities generated by established tools (Ross et al., 2021). Felicity had faced particular treatment uncertainty, having undergone hormone therapy to reduce the size of the tumour prior to surgery. She received a high score indicating chemotherapy and described this as “reassuring” despite recalling her mother’s gruelling experiences of the treatment:

I had, well, a score of twenty‐seven which was a no, no‐brainer really. That’s you at a high‐risk… I was glad that it was such a definitive result because I think had it been a really low result and we were saying, “well, we think that that low result is still okay, so maybe not bother having chemotherapy”, I think that would have been worse for me.

(Felicity)

Felicity’s result is represented as taking the decision about chemotherapy away (a ‘no‐brainer’). She explained that a result indicating the avoidance of chemotherapy would have been difficult for her, as she would have questioned whether a lower result following hormone treatment was reflective of “the actual true cancer”. Again, we observe a notion of there being a ‘correct’ result, as well as an orientation towards treatment; a recommendation to avoid treatment would have been questioned, but a high risk score was interpreted as ‘definitive’. This removal of choice was also articulated by Natasha who described her acceptance of chemotherapy following a ‘high‐risk’ result as a ‘Hobson’s choice’.^2^ For these women, proceeding to chemotherapy treatment was no longer experienced as just a felt imperative but as *mandated* by their Oncotype DX result; they now had a “clear line of action to take” (Berg, 1997, p. 505). This was linked to its perceived ‘advanced’ or ‘scientific’ status when compared with established tools and apparent provision of certainty.

The purported certainty offered by gene expression profiling was also positioned as removing choice by those whose results indicated an avoidance of chemotherapy. We have seen that many participants described an inclination towards accepting the treatment in the face of clinical uncertainty. This was to ‘do all they could’ to prevent a recurrence (Charles et al., 1998). Following their gene expression profiling result, those women with a low result discussed now feeling able to forgo chemotherapy, with the result providing ‘reassurance’ that this was the ‘right’ decision:

[Oncotype DX] has reassured me, definitely. I can say that, that not having chemo is a good idea.

(Zoe)

The information provided by Oncotype DX was positioned by Lois as crucial to her decision to eschew chemotherapy. Without this information, declining chemotherapy would not have been an option for her:

To be told medically that I didn’t need it then that was reassuring, rather than us saying “oh well, we’re borderline, no, we won’t have it, we’ll take the chances”. I don’t think I could have done that. I would have gone on with the chemo.

(Lois)

According to Lois, the test result rendered the avoidance of chemotherapy acceptable, an act which had until now been experienced as uncomfortable in the context of an imperative to treat (Charles et al., 1998: Bourgain et al., 2020). This was linked to its perceived superiority to other techniques by some, with Lois describing it as the ‘second opinion’ she needed, though Zoe described the result as “in addition to [NHS] Predict rather than instead of it”. These depictions of the influential role of Oncotype DX, as apparently determining chemotherapy decision‐making, seem straightforward. They accord with the perspective that genomic techniques allow patients to decline treatment, through their provision of additional and/or more ‘accurate’ information. These tools are therefore seen as able to reduce the problem of ‘overtreatment’. In the next section, we probe these accounts more closely. We demonstrate that though this ‘advanced’ technique seemed to sanction the avoidance of chemotherapy in some instances, the imperative to treat cancer persisted. This shaped women’s reflections on their choice, and for some their decision‐making with regards to other cancer treatments.

### ‘No other tests to try’: Diverse meanings of gene expression profiling

As discussed above, some patients valued gene expression profiling because of its perceived superiority over other techniques. However, though this discourse was common, and only a minority were able to provide specific details about the mechanism of the test. Some patients appreciated that the test assessed genetic or genomic information about their cancer, but understandings of how it did so were unclear. Interviewees’ cited results as being formulated using a blood sample (Julie), or by assessing growth of the tumour tissue in a laboratory (Valerie). Three participants articulated that such information did not matter to them, including Jane:

from the point of view of did I need to know how the genomics worked, I didn’t need to once I’d got the information that the test gave me… it’s a faith in scientists I suppose.

(Jane)

Here, Jane represents gene expression profiling testing as ‘scientific’. The test was similarly described by Bethany, who contrasted her “trust [in] the science” of gene expression profiling with the recommendation provided by her oncologist. The ‘scientific’ base for the test gave Jane and Bethany faith in the technique, despite a lack of awareness about its mechanism and evidence base. These accounts demonstrate the power of the high expectations surrounding genomic medicine to direct patients’ acceptance of Oncotype DX.

Significantly, Jane’s account indicates that the intricacies of the technique were not as important to her as the meaning and implications of the result for her treatment. It was what the technique enabled (i.e. for a chemotherapy decision to be made) that is positioned as key. This was also evident in the following interviews:

I was very pleased when I heard how the test worked because I thought “right, that’s fine because that makes it much more specific and that will make me feel I’m not making a hunch decision, I’m making a decision based on actual science, actually related to me” which felt far less like it was my responsibility… I was like “that feels like I’m going to make a doctor make a decision, not me.”

(Julie)

Interviewer What was so attractive about the [Oncotype DX] test that made you say “yes, I want this”?

Lois I think hoping that the [chemotherapy] decision wasn’t being left to us.

For these interviewees the result was not valued in and of itself, but welcomed because it enabled a deflection of the responsibility for chemotherapy decision‐making and the removal of interviewees from the ‘rock and the hard place’ in which they had been situated. As another example, Valerie positioned the result as ‘definite’ with regards to chemotherapy, not only because it provided ‘more information’ but because “This is it. There’s no other tests to try”.

In probing patient accounts, we go beyond the discourse of gene expression profiling being a more superior technique as an explanation for their faith in its result. Instead, we see a more complex engagement, whereby patients articulated having no choice but to accept the result in the face of an alternative where the burden of chemotherapy decision‐making would again fall to themselves. The allure and function of Oncotype DX within this context was discussed as more than simply providing specific information about their tumour. The technique held symbolic power because it represented a ‘final’ step on their journey towards a treatment decision. In the context of a treatment imperative, this decision had been experienced as a heavy responsibility, as emotionally and affectively fraught. Interpreting the technique’s function in this way, we can understand patients’ trust in Oncotype DX as a ‘leap of faith’ fuelled by wider regimes of hope surrounding novel biomedical developments (Franklin, 1997).

Aware of the difficulty of chemotherapy decision‐making, clinicians also recognised the benefits of the test to patients. This is despite some scepticism regarding the ‘added value’ it brought to their practice (Kerr et al., 2021). During one observation, the consultant described the role of Oncotype DX as “helping the patient to make‐up her mind” (Observation 2, June 2017). One oncologist said that patients think gene expression profiling “gives the answer” (Clinical oncologist 1), with another elaborating:

Patients often find [Oncotype DX] quite useful because it's difficult for them to make these decisions. So in a way having the test to tell you whether you should or shouldn’t have chemotherapy takes the difficulty away from you, and you just do what the test says. I think that's why a lot of patients quite like it.

(Medical oncologist 5)

Two further clinicians discussed the test’s value as going beyond the provision of clinical information. Gene expression profiling was represented as facilitating discussions about chemotherapy that had been more difficult in its absence. Medical oncologist 2 described that these ‘borderline’ patients were “always a trickier group beforehand to discuss the chemotherapy options with”. These ‘tricky’ conversations were re‐framed and re‐posed with the introduction of Oncotype DX into patient care. One medical oncologist explained that “when we request the test we try and establish with the patient as to what result would lead to what decision” (Medical oncologist 5). He and three other clinicians said these conversations were initiated because they would not want to perform the test, which was costly to the NHS but could also delay treatment, should the patient have already planned to either accept or refuse chemotherapy regardless of the result (Clinical oncologist 2, Medical oncologist 2 and Medical oncologist 4). Medical oncologist 5 explained that patients thus needed to formulate “a sort of predetermined decision” about whether to accept chemotherapy before having received their result, in some cases even before requesting the test. Whilst waiting for her results, Valerie and her husband determined that “if it was going to be a high reading… probably best benefit to do it. If it was low obviously not”. Dette’s clinician went as far as booking her in for chemotherapy in advance of the test result. This meant that when she did receive a high score there “wasn’t really much of a think‐about time, it was “that’s the result so that’s what’s going to happen now”. The prospect of testing prompted these interviewees to engage with decision‐making in alternative ways and from a different angle. They pondered hypothetical outcomes, shared these with others and delegated decision‐making to a future result.

Our analysis has shown that on the face of it, Oncotype DX appeared to make a rejection of the treatment imperative an acceptable choice, due to its positioning as a ‘scientific’, ‘advanced’ and ‘state of the art’ technique which reduced uncertainty. This echoes perspectives on genomic techniques which position them as providing the information required for clinicians and patients to make the ‘right’ decision about treatment. However, despite frequent portrayals of gene expression profiling as enabling some patients to decline treatment, when we explored these accounts in more depth, our interviews indicated that this could be experienced as tentative. For Julie, the removal of chemotherapy from her expected pathway remained uncomfortable and factored into her decisions about radiotherapy:

[I wanted to] make sure that I was still thinking “alright, well, what is the best course overall?” and not just try and short‐cut to the end of the process. Em, and in a way, maybe that is one of the reasons I decided to do the radiotherapy… I thought “right, I’ve escaped chemo, let’s tackle something tough”.

(Julie)

This accords with research outlining patient expectations that successful cancer treatment necessarily entails suffering (Bell, 2009). This discomfort could also be communicated by clinicians. Jane described accepting Zoladex injections (a hormone therapy) in addition to the standard Tamoxifen:

That was their belt and braces, if you want, using the same phraseology. But that was their way of saying, “we’ll give you this sort of beefed‐up hormone therapy since you’re not having the chemotherapy.”

(Jane)

Here, we see the disentangling of chemotherapy from cancer treatment positioned as something that needs to be compensated for. Though participants who decided not to proceed with chemotherapy may appear to have contravened a treatment imperative, their accounts reveal that it continued to overshadow their experiences and impact treatment pathways. An unsettling of the treatment imperative could also be experienced only temporarily. Five participants relayed a sense that because they had declined the treatment, their cancer may recur:

I guess if this recurs in the future, I’ll probably go “should I have done chemo at the time?” so, you know, that’s always going to happen… You know, so the problem is, there is no crystal ball and the test felt like it helped be a bit of a crystal ball, you know, and give me something to actually make a decision on, which was really good because I was in no fit state to do it myself.

(Julie)

Julie invokes the treatment imperative through her fear that without chemotherapy her cancer could recur, again portraying this in terms of a ‘right’ or ‘wrong’ decision. Fears about having made the ‘wrong’ decision were much more audible amongst those who declined chemotherapy. Despite acknowledging that gene expression profiling was ultimately unable to indicate the ‘right’ treatment, Julie credits it with allowing her to make this difficult choice by providing “something to make a decision on”, which Jane expressed as providing a ‘basis’ for rejecting chemotherapy. This aspect of the technique has, to date, been omitted from sociological discussions of gene expression profiling and wider work exploring the function of genomic techniques in treatment decision‐making. By attending to patient and clinician accounts of this novel test, we see the technique was not purely valued as an end in itself but as prompting more confronting forms of decision‐making work that, in the face of a strong imperative towards treatment, had hitherto been extremely difficult. Patients acknowledged the unpredictability of cancer and that in time it may emerge that they had made the ‘wrong’ choice. Nevertheless, Oncotype DX played an important function by attending to (though as we have seen, not always resolving) uncertainties generated by previous prognostic tools and allowing them to make this difficult decision. This was attested to by a Clinical Nurse Specialist who described that, though it could not resolve grey areas but only make them ‘smaller’, the technique allowed patients to “come up with answers that make them feel as comfortable as they can”. Though initially describing her ‘trust’ in its ‘science’ during her interview, Bethany ultimately described the inability of gene expression profiling to provide hoped‐for certainty about her cancer’s return. However, the technique remained highly valued. This is because in the context of an imperative towards treatment it allowed her to make a difficult decision at the time it was required and to look to a future beyond cancer:

I’m very grateful to have benefitted from [gene expression profiling]. Because basically, OK, I know I don’t know what the future holds and the tumour might come back and I might end up having to have chemotherapy. But if it’s felt that I don’t need it right now, then I can get on with life.

(Bethany)

## DISCUSSION AND CONCLUSION

Molecular techniques are now well‐established within UK breast oncology, entailing personalised rather than ‘one‐size‐fits‐all’ approaches to treatment (Yeo et al., 2014). We have shown that this complicates decision‐making in the clinic. While the existence of multiple and more effective therapeutic options can contribute to a treatment imperative (Shim et al., 2006), a paradox of genomic medicine is that the resulting recommendation may be to forgo a particular treatment. This may indeed be preferable in a context of overtreatment or where the treatment entails heavy side effects. Though purportedly providing greater certainty, techniques to determine the most appropriate treatment pathway can situate patients’ cancers within ‘grey areas’. In these cases, the clinicians we interviewed reported more explicitly sharing treatment decisions by soliciting patient preferences. This approach reflects contemporary shifts in doctor‐patient interaction, whereby ‘engaged patients’ are more willing and able to participate in clinical decision‐making (Timmermans, 2020). However, our participants resisted the opportunity to assume full responsibility for chemotherapy decision‐making. Though the character of medical authority has shifted across health care more broadly (Epstein & Timmermans, 2021), our participants privileged a clinical recommendation on this issue (see also Charles et al., 1998; Sinding et al., 2010). This, we argue, is shaped by the specific disease context. In cancer care, chemotherapy takes a heavy physical toll, yet the possibility of declining treatment can be uncomfortable for clinicians and patients or even perceived as ‘doing nothing’ (Charles et al., 1998). The endurance of the treatment imperative in the case of early breast cancer shapes but is also shaped by the introduction of gene expression profiling. Oncotype DX provides an opportunity to subvert the orientation towards treatment but only when authorised by an ‘advanced’ scientific technique. It also ensures that the ability to determine the ‘right’ treatment decision remains under the purview of medical authority.

Taking an approach which documents the mutual shaping of technologies and care practices (Berg, 1997; Kazimierczak, 2018), we have shown that established prognostic tools, clinician‐patient relationships and cultural imaginings of cancer and chemotherapy all co‐produce the treatment decision faced by patients with this type of early breast cancer. Following Bourgain et al. (2020), we argue that this configuration of techniques and practices has crafted a space for the introduction of gene expression profiling. The treatment imperative and the notion of a ‘right’ chemotherapy decision contributed to a need for reassurance in decision‐making and a desire for a more ‘accurate’ or ‘scientific’ technique. Oncotype DX was discussed by patients, interviewed in this study and by clinical staff, as providing much‐needed assistance. The technique was represented by them as a ‘technology of hope’ (Franklin, 1997). Some explicitly referenced its potential as a ‘state of the art’ or ‘advanced’ technique to transform patient care for the future, echoing wider discourses of promise and hype surrounding genomic techniques. However, their hopes for the technique were largely articulated at the level of their individual cancer trajectories, with their result having implications for their working and family lives and at the level of their emotional wellbeing. Interviewees described feeling ‘blessed’ and ‘relieved’ that they had been offered this test, so that this ‘impossible’ decision would no longer be theirs alone. It was this deeply emotive context that we propose rendered Oncotype DX particularly alluring to patients and, we argue, is crucial to consider when reflecting on the adoption of the technique in practice. Where examinations of the bioeconomies of novel techniques have often focussed on the commercial investments and public discourse that sustain their use in practice, our work follows Haase et al. (2015) in emphasising the more intimate contexts of the ‘private hopes, dreams and disappointments’ of those involved with these technologies in the everyday.

Related to the hope surrounding this technology is its symbolic value to patients and practitioners, beyond its provision of information about the cancer. Patients’ limited understanding of the test’s mechanism did not hinder their faith in it. It was valued by many because it was perceived as an ‘advanced technique’, with this sufficient to sanction the rejection of chemotherapy as a legitimate option. The technique played a role beyond its provision of clinical information for clinicians too. The enduring uncertainties of cancer meant that clinicians did not position Oncotype DX as straightforwardly resolving treatment decisions. However, its fallibilities did not preclude its use–like those interviewed by Bourgain et al. (2020), the shortfalls of the technique were controlled through its integration as an ‘additional tool’. Our research has demonstrated that some of the most significant work performed by the technique was not to supply a definitive recommendation but to provoke new conversations around the uncomfortable chemotherapy decision faced by patients and to provide patients (and clinicians) with confidence in the decisions that were eventually settled upon.

In contemporary biomedicine, patients are increasingly required to make sense of (sometimes unclear) genomic test results for the purposes of treatment decision‐making. In the context of early breast cancer, this can be extremely difficult for both patients and clinicians. Cultural narratives continue to portray cancer as a fatal disease and chemotherapy as ‘killer chemicals’ (Baszanger, 2012; Greenhalgh, 2017). This was attested to by interviewees in this study, who despite being given an optimistic prognosis described fear, uncertainty and sadness. Their encounters with Oncotype DX were situated within much longer diagnostic journeys involving an array of complex technologies, interventions and bioclinical collectives, the navigation of which required physical and emotional work. We opened this article by asking how the treatment imperative is experienced in light of gene expression profiling in early breast cancer. The answer is not straightforward. Oncotype DX provided some interviewees with a ‘basis’ to decline chemotherapy, enabling this to become a choice they could feel ‘comfortable’ with. However, in the face of inherent uncertainties surrounding cancer and its return, the relief provided by gene expression profiling could be short‐lived. The treatment imperative remained powerful to the extent that some patients even felt they must compensate for avoiding chemotherapy by undergoing other treatments. This challenges the idea that the ‘accuracy’ provided by genomic tools will allow clinicians to confidently recommend against a particular intervention, thus addressing the issue of overtreatment (see Katz et al., 2018). Such a perspective frames Oncotype DX as a neutral actor whose function is to uncover the ‘correct’ treatment recommendation to which patients will adhere. It ignores the multiple meanings of the technique, the investment of patient hopes and the wider cultural and organisational factors at play. By attending to these aspects, we have shown that the technique did not entirely mitigate the treatment imperative but could prompt its manifestation in other ways. Our work has drawn attention to the effects of novel genomic techniques beyond the clinical information they generate. We have shown how decision‐making tools work to constitute the difficult treatment decisions they are called to remedy but also emphasised their symbolic power. We have added a patient perspective to social scientific studies tracing the movement of novel genomic technologies into the clinic and shown the enduring importance of attention to the emotional and interactional aspects of treatment decision‐making in the genomic era.

## AUTHOR CONTRIBUTIONS

**Emily Ross:** Conceptualization (lead); Data curation (equal); Formal analysis (lead); Investigation (equal); Methodology (equal); Writing – original draft (lead); Writing – review & editing (lead). **Anne Kerr:** Conceptualization (supporting); Data curation (equal); Formal analysis (supporting); Funding acquisition (equal); Investigation (equal); Methodology (equal); Supervision (equal); Writing – original draft (supporting); Writing – review & editing (supporting). **Julia Swallow:** Conceptualization (supporting); Data curation (equal); Formal analysis (supporting); Investigation (equal); Methodology (equal); Writing – review & editing (supporting). **Choon Key Chekar:** Data curation (equal); Formal analysis (supporting); Investigation (equal); Methodology (equal); Writing – review & editing (supporting). **Sarah Cunningham‐Burley:** Data curation (equal); Formal analysis (supporting); Funding acquisition (equal); Investigation (equal); Methodology (equal); Supervision (equal); Writing – review & editing (supporting).

## Data Availability

Some of the data that support the findings of this research are available upon request from the Restricted Access Data Repository—Leeds, at https://doi.org/10.5518/1247.

## ENDNOTES

^1^
Though some cancer types, including pancreatic, remain under‐researched and survival continues to be poor.

^2^
A proverb describing a forced course of action that presents itself as a free choice.

## References

[shil13637-bib-0001] Ackerman, S. L. (2022). Promising precision medicine: How patients, clinicians and caregivers work to realize the potential of genomics‐informed cancer care. New Genetics & Society, 41(3), 196–215. 10.1080/14636778.2021.1997577

[shil13637-bib-0002] Armstrong, N. (2021). Overdiagnosis and overtreatment: A sociological perspective on tackling a contemporary healthcare issue. Sociology of Health & Illness, 43(1), 58–64. 10.1111/1467-9566.13186 32964516

[shil13637-bib-0003] Baszanger, I. (2012). One more chemo or one too many? Defining the limits of treatment and innovation in medical oncology. Social Science & Medicine, 75(5), 864–872. 10.1016/j.socscimed.2012.03.023 22658622

[shil13637-bib-0004] Bell, K. (2009). ‘If it almost kills you that means it's working!’ Cultural models of chemotherapy expressed in a cancer support group. Social Science & Medicine, 68(1), 169–176. 10.1016/j.socscimed.2008.10.023 19010578

[shil13637-bib-0005] Berg, M. (1997). Rationalizing medical work: Decision‐support techniques and medical practices. MIT Press.

[shil13637-bib-0006] Borgstrom, E. , Cohn, S. , & Driessen, A. (2020). ‘We come in as “the nothing”’: Researching non‐intervention in palliative care. Medicine Anthropology Theory, 7(2), 202–213. 10.17157/mat.7.2.769

[shil13637-bib-0007] Bourgain, C. , Pourtau, L. , Mazouni, C. , Bungener, M. , & Bonastre, E. J. (2020). Imperfect biomarkers for adjuvant chemotherapy in early stage breast cancer with good prognosis. Social Science & Medicine, 246, 112735. 10.1016/j.socscimed.2019.112735 31869667

[shil13637-bib-0008] Bourret, P. , Keating, P. , & Cambrosio, A. (2011). Regulating diagnosis in post‐genomic medicine: Re‐aligning clinical judgment? Social Science & Medicine, 73(6), 816–824. 10.1016/j.socscimed.2011.04.022 21664021

[shil13637-bib-0009] Casper, M. J. , & Morrison, D. R. (2010). Medical sociology and technology: Critical engagements. Journal of Health and Social Behavior, 51(1), S120–S132. 10.1177/0022146510383493 20943577

[shil13637-bib-0010] Charles, C. , Whelan, T. , Gafni, A. , Reyno, L. , & Redko, C. (1998). Doing nothing is no choice: Lay constructions of treatment decision‐making among women with early‐stage breast cancer. Sociology of Health & Illness, 20(1), 71–95. 10.1111/1467-9566.00081

[shil13637-bib-0011] Charmaz, K. (2006). Constructing grounded theory: A practical guide through qualitative analysis. Sage Publications.

[shil13637-bib-0012] Chekar, C. K. , Kerr, A. , & Swallow, J. (2022). Translations and transformations in patienthood: Cancer in the post‐genomics era. University of Leeds. [Dataset]. 10.5518/1247

[shil13637-bib-0013] Clarke, A. E. , Mamo, L. , Fosket, J. R. , Fishman, J. R. , & Shim, J. K. (2010). Biomedicalization: Technoscience, health and illness in the U.S. Duke University Press.

[shil13637-bib-0014] Crolley, V. E. , Marashi, H. , Rawther, S. , Sirohi, B. , Parton, M. , Graham, J. , Vinayan, A. , Sutherland, S. , Rigg, A. , Wadhawan, A. , Harper‐Wynne, C. , Spurrell, E. , Bond, H. , Raja, F. , & King, J. (2020). The impact of Oncotype DX breast cancer assay results on clinical practice: A UK experience. Breast Cancer Research and Treatment, 180(3), 809–817. 10.1007/s10549-020-05578-6 32170635 PMC7103011

[shil13637-bib-0015] Davis, C. (2015). Drugs, cancer and end‐of‐life care: A case study of pharmaceuticalization? Social Science & Medicine, 131, 207–214. 10.1016/j.socscimed.2014.12.007 25533871 PMC4376382

[shil13637-bib-0016] Day, S. , Coombes, R. C. , Mcgrath‐Lone, L. , Schoenborn, C. , & Ward, H. (2017). Stratified, precision or personalised medicine? Cancer services in the ‘real world’ of a London hospital. Sociology of Health & Illness, 39(1), 143–158. 10.1111/1467-9566.12457 27460935

[shil13637-bib-0017] del Vecchio Good, M.‐J. , Good, B. J. , Schaffer, C. , & Lind, S. E. (1990). American oncology and the discourse on hope. Culture, Medicine and Psychiatry, 14(1), 59–79. 10.1007/bf00046704 2340733

[shil13637-bib-0018] Epstein, S. , & Timmermans, S. (2021). From medicine to health: The proliferation and diversification of cultural authority. Journal of Health and Social Behavior, 62(3), 240–254. 10.1177/00221465211010468 34528483

[shil13637-bib-0019] Franklin, S. (1997). Embodied progress: A cultural account of assisted conception. Routledge.

[shil13637-bib-0020] Greenhalgh, T. (2017). Adjuvant chemotherapy: An autoethnography. Subjectivity, 10(4), 340–357. 10.1057/s41286-017-0033-y

[shil13637-bib-0021] Haase, R. , Michie, M. , & Skinner, D. (2015). Flexible positions, managed hopes: The promissory bioeconomy of a whole genome sequencing cancer study. Social Science & Medicine, 130, 146–153. 10.1016/j.socscimed.2015.02.016 25697637 PMC4363274

[shil13637-bib-0022] Heath, I. (2014). Role of fear in overdiagnosis and overtreatment—An essay by Iona Heath. BMJ British Medical Journal, 349(aug06 2), g6123. 10.1136/bmj.g6123 25954986

[shil13637-bib-0023] Katz, S. J. , Jagsi, R. , & Morrow, M. (2018). Reducing overtreatment of cancer with precision medicine: Just what the doctor ordered. JAMA, 319(11), 1091–1092. 10.1001/jama.2018.0018 29470568

[shil13637-bib-0024] Kazimierczak, K. A. (2018). Clinical encounter and the logic of relationality: Reconfiguring bodies and subjectivities in clinical relations. Health, 22(2), 185–201. 10.1177/1363459316688521 28401816

[shil13637-bib-0025] Kazimierczak, K. A. , & Skea, Z. (2015). ‘I've used the word cancer but it's actually good news’: Discursive performativity of cancer and the identity of urological cancer services. Sociology of Health & Illness, 37(3), 340–354. 10.1111/1467-9566.12192 25847531

[shil13637-bib-0026] Keating, P. , Cambrosio, A. , & Nelson, N. C. (2016). “Triple negative breast cancer”: Translational research and the (re)assembling of diseases in post‐genomic medicine. Studies in History and Philosophy of Science Part C: Studies in History and Philosophy of Biological and Biomedical Sciences, 59, 20–34. 10.1016/j.shpsc.2016.05.003 27235853

[shil13637-bib-0027] Kerr, A. , Chekar, C. , Ross, E. , Swallow, J. , & Cunningham‐Burley, S. (2021). Personalised cancer medicine: Future crafting in the genomic era. Manchester University Press. 33555770

[shil13637-bib-0028] Kerr, A. , Swallow, J. , Chekar, C. K. , & Cunningham‐Burley, S. (2019). Genomic research and the cancer clinic: Uncertainty and expectations in professional accounts. New Genetics & Society, 38(2), 222–239. 10.1080/14636778.2019.1586525 31156350 PMC6519890

[shil13637-bib-0029] Kreiner, M. J. , & Hunt, L. M. (2014). The pursuit of preventive care for chronic illness: Turning healthy people into chronic patients. Sociology of Health & Illness, 36(6), 870–884. 10.1111/1467-9566.12115 24372285 PMC4074448

[shil13637-bib-0030] Loncaster, J. , Armstrong, A. , Howell, S. , Wilson, G. , Welch, R. , Chittalia, A. , Valentine, W. J. , & Bundred, N. J. (2017). Impact of Oncotype DX breast Recurrence Score testing on adjuvant chemotherapy use in early breast cancer: Real world experience in Greater Manchester, UK. European Journal of Surgical Oncology, 43(5), 931–937. 10.1016/j.ejso.2016.12.010 28111076

[shil13637-bib-0031] Marshall, D. A. , Deal, K. , Bombard, Y. , Leighl, N. , Macdonald, K. V. , & Trudeau, M. (2016). How do women trade‐off benefits and risks in chemotherapy treatment decisions based on gene expression profiling for early‐stage breast cancer? A discrete choice experiment. BMJ Open, 6(6), e010981. 10.1136/bmjopen-2015-010981 PMC4893875 27256091

[shil13637-bib-0032] Mrig, E. H. , & Spencer, K. L. (2018). Political economy of hope as a cultural facet of biomedicalization: A qualitative examination of constraints to hospice utilization among U.S. end‐stage cancer patients. Social Science & Medicine, 200, 107–113. 10.1016/j.socscimed.2018.01.033 29421457

[shil13637-bib-0033] National Institute for Health and Care Excellence . (2018). Tumour profiling tests to guide adjuvant chemotherapy decisions in early breast cancer. [Online]. Retrieved August 10, 2022, from https://www.nice.org.uk/guidance/dg34

[shil13637-bib-0034] Pak, L. M. , & Morrow, M. (2022). Addressing the problem of overtreatment in breast cancer. Expert Review of Anticancer Therapy, 22(5), 535–548. 10.1080/14737140.2022.2064277 35588396 PMC9448354

[shil13637-bib-0035] Ross, E. , Swallow, J. , Kerr, A. , Chekar, C. K. , & Cunningham‐Burley, S. (2021). Diagnostic layering: Patient accounts of breast cancer classification in the molecular era. Social Science & Medicine, 278, 113965. 10.1016/j.socscimed.2021.113965 33940433 PMC8146724

[shil13637-bib-0036] Ross, E. , Swallow, J. , Kerr, A. , & Cunningham‐Burley, S. (2019). Online accounts of gene expression profiling in early‐stage breast cancer: Interpreting genomic testing for chemotherapy decision making. Health Expectations, 22(1), 74–82. 10.1111/hex.12832 30387238 PMC6351409

[shil13637-bib-0037] Samuel, G. N. , & Farsides, B. (2017). The UK’s 100,000 Genomes Project: Manifesting policymakers’ expectations. New Genetics & Society, 36(4), 336–353. 10.1080/14636778.2017.1370671 29238265 PMC5706982

[shil13637-bib-0038] Shim, J. K. , Russ, A. J. , & Kaufman, S. R. (2006). Risk, life extension and the pursuit of medical possibility. Sociology of Health & Illness, 28(4), 479–502. 10.1111/j.1467-9566.2006.00502.x 16669809

[shil13637-bib-0039] Shim, J. K. , Russ, A. J. , & Kaufman, S. R. (2008). Late‐life cardiac interventions and the treatment imperative. PLoS Medicine, 5(3), e7. 10.1371/journal.pmed.0050007 18318595 PMC2270285

[shil13637-bib-0040] Sinding, C. , Hudak, P. , Wiernikowski, J. , Aronson, J. , Miller, P. , Gould, J. , & Fitzpatrick‐Lewis, D. (2010). “I like to be an informed person but…” negotiating responsibility for treatment decisions in cancer care. Social Science & Medicine, 71(6), 1094–1101. 10.1016/j.socscimed.2010.06.005 20633970

[shil13637-bib-0041] Spencer, K. L. , Mrig, E. H. , & Talaie, A. K. (2022). The many faces of medical treatment imperatives: Biopower and the cultural authority of medicine in late‐life treatment decisions in the United States. Sociology of Health & Illness, 44(4–5), 781–797. 10.1111/1467-9566.13459 35243659

[shil13637-bib-0042] Steinberg, D. L. (2015). The bad patient: Estranged subjects of the cancer culture. Body & Society, 21(3), 115–143. 10.1177/1357034x15586240

[shil13637-bib-0043] Sulik, G. A. (2009). Managing biomedical uncertainty: The technoscientific illness identity. Sociology of Health & Illness, 31(7), 1059–1076. 10.1111/j.1467-9566.2009.01183.x 19619153

[shil13637-bib-0044] Tate, A. (2022). Death and the treatment imperative: Decision‐making in late‐stage cancer. Social Science & Medicine, 306, 115129. 10.1016/j.socscimed.2022.115129 35717824 PMC10772987

[shil13637-bib-0045] Timmermans, S. (2020). The engaged patient: The relevance of patient–physician communication for twenty‐first‐century health. Journal of Health and Social Behavior, 61(3), 259–273. 10.1177/0022146520943514 32723112

[shil13637-bib-0046] Timmermans, S. , & Berg, M. (2003). The practice of medical technology. Sociology of Health & Illness, 25(3), 97–114. 10.1111/1467-9566.00342 14498932

[shil13637-bib-0047] Timmermans, S. , Tietbohl, C. , & Skaperdas, E. (2017). Narrating uncertainty: Variants of uncertain significance (VUS) in clinical exome sequencing. BioSocieties, 12(3), 439–458. 10.1057/s41292-016-0020-5

[shil13637-bib-0048] Viney, W. , Day, S. , Bruton, J. , Gleason, K. , Ion, C. , Nazir, S. , & Ward, H. (2022). Personalising clinical pathways in a London breast cancer service. Sociology of Health & Illness, 44(3), 624–640. 10.1111/1467-9566.13441 35143700 PMC9303177

[shil13637-bib-0049] Yeo, B. , Turner, N. C. , & Jones, A. (2014). An update on the medical management of breast cancer. BMJ British Medical Journal, 348(jun09 1), g3608. 10.1136/bmj.g3608 24912480

